# Integrating omics atlas in health informatics system design-an opinion article

**DOI:** 10.3389/fdgth.2024.1374359

**Published:** 2024-05-09

**Authors:** Irene Suilan Zeng

**Affiliations:** ^1^ Department of Biostatistics and Epidemiology, Auckland University of Technology, Auckland, New Zealand; ^2^ School of Clinical Science, Faculty of Health and Environmental Sciences, Auckland University of Technology, Auckland, New Zealand

**Keywords:** integrated system-biology, integrated health information system, precision medicine, personalized medicine, centralized integrated system

## Introduction

Human health includes 30%–40% of clinical determinants, the rest of the determinants are genetic, environmental, social and behavioural (1). The genetic and environmental information that contributes to 10%–30% of human health determinants is recorded within an individual's system-biology (2, 3), the multiple biological information entities from the whole collections of genes/proteins/metabolites. Precision and personalized health informatics are scientific areas that utilize system-biology to improve an individual's health. These areas could reach their full potential by designing an architecture of these multiple systems to support the decision-making process in diagnosis, monitoring, and prevention. The recent publication “INTUITION: a data platform to integrate human epilepsy clinical care and support for discovery” (4) has provided an excellent frontier example of the system-biology information, integrated with clinical information. This opinion article responds to this call by suggesting a centralized system with translation and other components that could make these types of integrated systems become utilized in real practice.

## The human proteome project

The importance and potentials of the system-biology, including proteomics and other omics data in the integrated system, emerge from its final translational phases of the entire pathway, which starts from discovery, prioritization, design, and optimization (5). Discovered and optimized results translated into a health context will enrich their clinical utilization. For example, better clinical pathways for diagnosis, treatment, and prognosis. Precision medicine will take its full potential when the integrated system-biology and health informatics system is established. The instrumental role of system-biology in precision medicine relies on a robust system that can integrate the translational outputs into the daily clinical function; vice versa, the daily clinical functional data will accelerate and improve the precision of the system-biology discovery. To facilitate the complexities of data pathways, data flows, and integration, we need to design a system that will optimize patient outcomes.

After the Human Genome Project (HGP) was completed in year 2003 (6), the HUPO Council started the Human Proteome Project (HPP) (7) in 2009 (8). It aims to map the entire human proteome to understand human biology at the cellular level and establish a foundation for diagnosis, prognostic, therapeutic and preventive medical applications. Gene-centric human proteome mapping has been complemented by in-depth studies of mapping proteomes with physiologic and pathologic states. Both HGP and HPP provided enriched publicly available data for basic and clinical scientists. There were also other emerging individual projects; for example, the Human Protein Atlas project (HPA) has generated a tissue-based map of the human proteome based on transcriptome data, antibody staining and expression of RNA (9). The omics data are ready to be integrated with health informatics data.

## Recent enhancement in the multi-omics data integration

The recent enhancement in multi-omics data integration provides relevant functions for translational medicine and new components for integrated health informatics. These tools and methods propose multimodal integration, including supervising and non-supervising approaches, using Frequentist and Bayesian methods from bulk or single cell omics. The functionalities of these tools can be streamlined into:

(1) Disease subtyping and classification, where the patients will be classified at their molecular and multi-omics levels. The discovered subtypes and classification will effectively enhance treatments for patients. Examples of these methods/tools are Patient-specific data fusion (PSDF), iclusters, and Pathway Recognition Algorithm using Data Integration on Genomic Models (PARADIGM).
(2) Prediction of biomarkers for diagnosis and prognosis, where identified multi-omics markers with genotypes and other patient predictors are included in statistical prediction models for risk and clinical outcomes.
(3) Disease biology insight (10) can be obtained through the multi-omics interaction networks (11) and biological pathways to reveal their regulatory processes. Understanding detailed disease mechanisms through multi-omics will help diagnose and derive innovative treatments.
(4) Drug response prediction and repurposing (12) through drug and multi-omics interaction networks (e.g., genes and proteins) (13).

These abovementioned streams use one or a combination of these typical methods: multiple data integration (14), network (15) and cluster approach (16, 17), patient fusion-based (18, 19), similarity-based (20) and other multivariate methods (e.g., Factor analysis, multi-block partial least square regression) (21).

As an example, “IntegratedLearner” (22) is a recent integrated model using a fully Bayesian Ensemble approach for classification and prediction through a multi-layer omics dataset controlling for single-layer omics bias. “IntegratedLearner” uses two-stage feature selection, allowing adjustment for confounding (e.g., environment, lifestyle) effects in both cross-sectional and longitudinal data. GLUE (23) is another recently developed tool for single cell multi-omics data integration. Utilizing prior biological knowledge guidance, it models the regulatory interactions across omics layers.

Multi-omics has many more applications in oncology via different machine learning methods for precision oncology in clinical practice (12). Its utilizations include data integration, statistical analysis, and the creation of Artificial Intelligence tools. Integrated approaches allow for an amplified view of genetic, biochemical, metabolic, proteomic, and epigenetic processes underlying cancer conditions that cannot be comprehended using single-omics approaches.

## Recent emerging example, “INTUITION” and integrated system-biology health informatic system

INTUITION (4) is a deidentified multimodal database platform that integrates system-biology omics data, neuroimaging, electrophysiology (EEG), neuropsychology, cellular (histology), and clinical data. Its system design and user interface include data upload/download, transformation, and data viewers with visualization. The storage units of the system comprise a file store, database, and remote storage. The purpose of the INTUITION platform is to provide an integrated understanding of information curated from biological, functional, clinical, and health data to elucidate the complex mechanism of epilepsy for better treatments. It has utilized the recent breakthrough of system-biology omics data curated from the removal of brain tissue cells. These integrations between different models include the spatial mapping between brain tissues and the electrode position, 3D imaging and omics. Its inventory management of proteins also facilitates the linkage between protein quantities, EEG electrodes, EEG quantified results, and MRI coordinates.

It is an advanced integrated informatics system but has not included human translational results and is designed for research purposes. An integrated system designed for routine clinical practice with a transition to public health will need tools and platforms of translational function, interactions between machine and human data feeds, and data flows with centralized and multiple entry points. Adding translational platforms could be the solution to make the integrated system work within the routine clinical practice.

**Figure 1:**
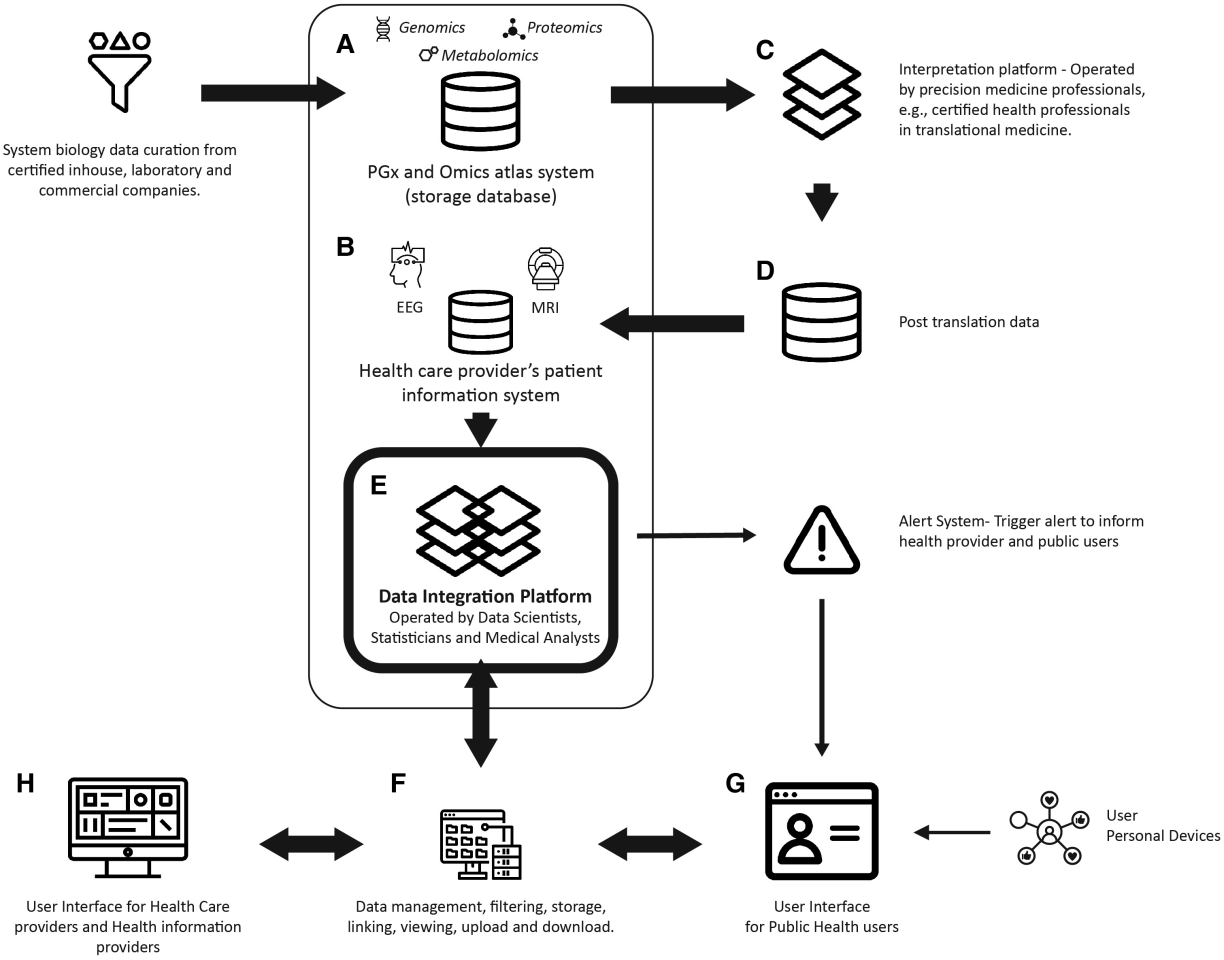
An integrated system-biology and health informatics centralized system.

The components of the system-biology and clinical information integrated system could include the following components described in Table 1, with the interpretation platform being the human-machine interaction portal. Figure 1 visualizes this kind of centralized system, with the potential to add on other home and personal devices, e.g., a neuropsychological assessment (24), a home environmental sensor for motion such as fall detection (sense4safety) (25), and advanced personalized medicine tests, e.g., pharmacogenetic tests (Figure 1. component G). In a centralized, integrated system-biology health informatic system, all data information is entered through different entry points from health providers of central and regional units (Figure 1. component H) and then stored in a central data portal (26). The primary function of the centralized system is to integrate the translational summary derived from system-biology analysis with routine clinical information (Figure 1. component E).

**Table 1:** The components of an integrated system-biology health informatics system.

Components	Functions
1. System-biology data, including test results using high throughput biotechnology of omics (e.g., Figure 1. component A).	Data upload, a platform for data management, filtering, storage, linkage, viewing, and data download
2. Interpretation platforms, including laboratory summary reports, visualizations, and algorithms of mappings and translations from the system-biology data (e.g., Figure 1. component C)	Interpretational report and summary, visualization, and algorithms for mapping
3. Clinical data, including clinical and any health information provided by health care providers, pharmacists, and other health information suppliers (e.g., post-translational system-biology results) (e.g., Figure 1. components B and D).	Multimodal databases
4. Health care provider portals: platforms that facilitate health care providers’ gathering and extracting patients’ information to assist their diagnosis, prescription, and next-step referral (e.g., Figure 1. H component). (e.g., Figure 1. component H).	These portals will also distribute patients’ information related to patients’ health conditions to a higher-level centralized system.
5. User/patient portals: platforms that facilitate users’/patients’ gathering of daily information and help them monitor and understand their health conditions (e.g., Figure 1. component G).	Personal devices can attach to these users’ portals.
6. Information feed design: the directions and pathways for gathering, centralizing, and distributing the data module.	System design
7. Integration tools that include high-level software used to link data from different portals and central databases (e.g., Figure 1. component E).	It is the core and primary function of the system to perform data integration
8. Security protection tools to prevent data confidentiality and information breaches (e.g., Figure 1. within components B, F, G, and H).	Detaining patient identities and providing system security functions.

Based on the Common Data Models (CDMS) design of a centralized system for data storage, linkage, and distribution for research and health surveillance (26), an integrated system has a core data portal with different data entry points, multimodal data storage components (e.g., Figure 1. components A and B), data processing platforms for editing/filtering and viewing (Figure 1. component F), and platforms to translate results from system-biology tests (Figure 1. component C). As a function of the CDMS, it will also provide a trigger system to send alarms and notifications to health providers and end-users, including allergy reactions, abnormal drug responses, and adverse events. The user interfaces are designed for both healthcare providers and public users of health services.

## Conclusion

An integrated system-biology health informatics system will enhance the full potential of personalized and precision medicine to deliver promising treatment as expected. The complex integration between system-biology and health information requires consideration of optimal infrastructure, security and privacy protection, linkage precision, storage capacities and inequities. The challenge from multi data modality integration in system-biology exists in missing data, inter-omics variations, and large data volume.

Some potential solutions could be considered in the integration:

1. Use standardization and data stewardship to reduce inter-omics variation and optimize data integration.
2. Work with global authorities and experts, such as Health Level Seven International (HL7) (27).
3. Consider co-designing with different end-users (live experience patients and care providers).
4. Consider universal informatics frameworks, such as the FAIR (findable, accessible, interoperable, and reusable) framework (28) to achieve an optimal infrastructure.
5. Set up translational medicine guidelines for data integration, methods, and interpretation for security, privacy protection and better linkage precision (11).
6. Encourage vertical collaborations between basic scientists, clinical scientists, and health professionals to improve interdisciplinary translations.
7. Use Multi-modality design in the multi-omics data integration, Bayesian methods/tools utilizing prior knowledge for coping with missing information and inter-omics variations.

Despite the complexity of integrating system-biology information into routine health informatics, the technologies developed today within these related disciplinary areas are well prepared to integrate them.
